# Further Insights Into Caprine Arthritis Encephalitis (CAE): The Current Status of Seroprevalence Among Small Ruminants in Two Selected States of Peninsular Malaysia

**DOI:** 10.21315/tlsr2021.32.2.6

**Published:** 2021-06-29

**Authors:** Bura Thlama Paul, Hamza Abdirahman Hashi, Nurul Najwa Burhannuddin, Eric Lim Teik Chung, Faez Firdaus Abdullah Jesse, Mohd Azmi Mohd Lila, Abd Wahid Haron, Azlan Che Amat, Yusuf Abba, Arsalan Maqbool, Khaleeq ur Rehman Bhutto, Kamarulrizal Mat Isa, Nur Azhar Amira, Mohammad Naji Odhah, Idris Umar Hambali, Mohd Jefri Norsidin

**Affiliations:** 1 Institute of Tropical Agriculture and Food Security, Universiti Putra Malaysia, 43400 Serdang, Selangor, Malaysia; 2 Department of Veterinary Clinical Studies, Faculty of Veterinary Medicine, Universiti Putra Malaysia, 43400 Serdang, Selangor, Malaysia; 3 Veterinary Teaching Hospital, Faculty of Veterinary Medicine, University of Maiduguri, PMB 1069, Maiduguri 600233, Borno State, Nigeria; 4 Department of Animal Science, Faculty of Agriculture, Universiti Putra Malaysia, 43400 Serdang, Selangor, Malaysia; 5 Department of Pathology and Microbiology, Faculty of Veterinary Medicine, Universiti Putra Malaysia, 43400 Serdang, Selangor, Malaysia; 6 Department of Veterinary Pathology, Faculty of Veterinary Medicine, University of Maiduguri, PMB 1069, Maiduguri 600233, Borno State, Nigeria; 7 Department of Veterinary Clinical Studies, Faculty of Veterinary Medicine, University Malaysia Kelantan, 16100 Kota Bharu, Kelantan Malaysia; 8 Department of Veterinary Public Health and Preventive Medicine, University of Maiduguri, PMB 1069, Maiduguri 600233, Borno State, Nigeria

**Keywords:** CAEV, Goats, Sheep, Seroprevalence, Contributing Factors, Peninsular Malaysia, CAEV, Kambing, Bebiri, Kadar Kelaziman Serologi, Faktor Penyumbang, Semenanjung Malaysia

## Abstract

Caprine arthritis-encephalitis virus (CAEV) is a member of the genus lentivirus causing caprine arthritis-encephalitis (CAE), a chronic inflammatory condition affecting the lungs, joints, udder and central nervous system of small ruminants such as sheep and goats. CAE is distributed worldwide and is recognised as a significant cause of morbidity and decreased milk production in dairy goats. Earlier studies highlighted the clinicopathological features and supplied preliminary serological evidence for the existence of CAE among selected goat herds in Malaysia. Therefore, this study aims to provide further insights into the seroprevalence and contributing factors of CAE among sheep and goat herds in two states of Peninsular Malaysia. The blood samples and biodata were randomly collected from a total of 262 individual sheep (40) and goat (222) in seven smallholder farms. Blood sera were tested for specific anti-CAEV antibodies using Qayee-Bio CAEV sandwich-ELISA test kits according to standard procedures. Our results of the study revealed 21.4% (95% CI: 15.8–28.6) apparent and 20.6% (95% CI: 14.5–27.8) true seroprevalence with significant differences (*p* < 0.05) in seroconversion rates between the states, farms, production systems and breeds of small ruminants. The prevalence of CAE in the Malaysian Peninsular is a potential threat to the small ruminant industry and developing agricultural economy. Further studies are required to determine the genetic characteristics, distribution and risk factors of CAEV for effective prevention and control in Malaysia.

## Abstract (fr)

Virus Kaprin Artritis Ensefalitis (CAEV) merupakan ahli kumpulan dalam genus virus lentivirus dimana akan menyebabkan penyakit kaprin artritis ensefalitis (CAE) di mana penyakit ini akan menyebabkan keradangan kronik pada paru-paru, sendi, kelenjar mamari dan sistem saraf pusat bagi haiwan ruminan kecil seperti bebiri dan kambing. CAE telah merebak ke seluruh dunia dan penyakit ini akan menyebabkan penularan wabak pada kadar morbiditi yang tinggi dan mengurangkan kuantiti penghasilan susu bagi kambing tenusu. Kajian terdahulu memberi penekanan kepada dapatan klinikal patologi dan data bukti serologi kewujudan penyakit CAE dalam kalangan gerompok kambing di Malaysia. Maka, kajian kini bertujuan memberikan pendedahan awal berkaitan kadar kelaziman serologi dan faktor yang menyumbang penyakit CAE dalam kalangan bebiri dan kambing bagi dua negeri di Semanjung Malaysia. Sampel darah dan data biologi telah dikumpulkan secara rawak dengan jumlah sampel 262 ekor (40 ekor bebiri dan 222 ekor kambing) daripada tujuh buah ladang peternak kecil. Serum yang telah dikumpul diuji dengan antibodi spesifik anti-CAEV dengan menggunakan prosedur piawai kit elisa daripada Qayee-Bio CAEV. Keputusan kajian ini menunjukkan kadar kelaziman serologi 21.4% (95% CI: 15.8–28.6) jelas dan 20.6% (95% CI: 14.5–27.8) kadar kelaziman benar dengan perbezaan ketara (*p* < 0.05) dalam kadar perubahan kelaziman serologi antara negeri, ladang, sistem produksi dan baka haiwan ruminan kecil. Dengan wujudnya kelaziman penyakit CAE di Semenanjung Malaysia ini akan menyumbang kepada kemungkinan ancaman negatif terhadap industri ruminan kecil dan sektor ekonomi dalam bidang penternakan. Lebih banyak kajian diperlukan untuk menentukan ciri genetik virus penyebab penyakit ini, taburan penyakit dan faktor penyumbang bagi CAEV supaya dapat mengadakan kawalan dan pencegahan efektif bagi penyakit ini di Malaysia.

**Highlights**

- Caprine arthritis-encephalitis (CAE) is a chronic inflammatory disease affecting the lungs, joints, udder and central nervous system of sheep and goats and is currently an emerging disease in the Tropics, particularly in Malaysia.
- The results of our study revealed 21.4% (95% CI: 15.8–28.6) apparent and 20.6% (95% CI: 14.5–27.8) true seroprevalence with significant differences (*p* < 0.05) in seroconversion rates between the states, farms, production systems and breeds of small ruminants from two states of Peninsular Malaysia.
- Further holistic studies are required to determine the genetic characteristics, distribution and risk factors of CAE among the small ruminant population in Malaysia for the implementation of effective prevention and control strategies.

## INTRODUCTION

Caprine arthritis-encephalitis virus (CAEV) and the closely related Maedi-Visna (MV) are known as small ruminant lentiviruses (SRLVs) [Office of International Epizootics (OIE) 2018]. CAEV, first discovered in 1974 as a significant cause of chronic inflammatory disease in goats, sheep and other related ruminants (Reina *et al*. 2006; Tu *et al*. 2017). Perinatal transmission of CAEV occurs *in-utero*, through direct vaginal contact during birth, accidental ingestion of colostrum from the infected dam, or exposure to respiratory and salivary secretions during neonate licking after birth (Rowe & East 1997; Andrioli *et al*. 2006; Souza *et al*. 2013). Long-term close contact with respiratory, salivary or postpartum lochia secretions of CAEV-infected dam, marking, ear tagging and open dehorning operations can increase the likelihood of post-natal transmission among goats (Pisoni *et al*. 2007). Furthermore, the presence of virus-infected cells in oestrus mucus, preputial swabs and epididymis of infected animals also favours venereal transmission (Rowe & East 1997; Brajon *et al*. 2012). The development of persistent, mononuclear inflammatory lesions in the lungs, joints udder and central nervous system causes morbidity and decreased milk production (Lilenbaum *et al*. 2007). Although affected animals are mostly asymptomatic persistent carriers (Adams & Gorham 1986), the clinical disease causes dyspnoea due to emaciation and progressive pneumonia in sheep or polyarthritis and mastitis in goats (Lilenbaum *et al*. 2007).

The CAE infection has been reported worldwide and is highly prevalent in countries that practice intensive production of dairy goats (Rowe & East 1997) such as Australia (Cutlip *et al*. 1992), United States (Greenwood 1995), Alexandria (Baraka *et al*. 2018), Canada, Switzerland, Norway, France (Lofstedt *et al*. 1988) and Jordan (Al-Qudah *et al*. 2006). On the other hand, the prevalence of CAE is generally lower in countries that rely on importation of livestock such as Saudi Arabia (Alluwaimi *et al*. 1990), India (Waseem *et al*. 2015), Thailand (Lin *et al*. 2011), Brazil (Bandeira *et al*. 2009) and Malaysia (Jesse *et al*. 2018). The routine diagnosis of CAE depends on clinicopathological evaluation and various laboratory methods such as indirect serology testing and direct molecular assays (Barquero *et al*. 2011; Barquero *et al*. 2013). While the search for a gold-standard diagnostic technique continues, the Office of International Epizootics (OIE) had in 2004 recommended the enzyme-linked immunosorbent assay (ELISA) and agar gel immunodiffusion test (AGIDT) for the diagnosis of SRLVs (Barquero *et al*. 2013; OIE 2018). More sensitive but rarely used diagnostic techniques in molecular surveillance of CAEV include the western blot, radioimmunoprecipitation (Peterhans *et al*. 2004; Tu *et al*. 2017), isothermal amplification and polymerase chain reaction (PCR) assays (Barquero *et al*. 2013).

SRLVs cause reduced productivity and delayed maturity in sheep and goats worldwide (Greenwood 1995; Konishi *et al*. 2016). Caprine arthritis encephalitis is a life-long infection which produces chronic and progressive arthritis, pneumonia, weight loss, encephalomyelitis, and indurative mastitis in small ruminants (Kaba *et al*. 2012). The effects of CAEV on productivity is due to abnormalities in the udder, lungs, and kidneys (Noordin *et al*. 2010) which leads to reduction in milk production and increased culling of animals, especially when the herd prevalence of infection is high (Ling *et al*. 2013). The pioneer work of Noordin *et al*. (2010) described the clinical signs and lesions of CAEC in goat in Malaysia. Following the report of Noordin and co-workers, Ling *et al*. (2013) described the neurological signs, postmortem and histopathological features of CAE infection in a goat kid presented to the Large Animal Ward, University Veterinary Hospital (UVH), Universiti Putra Malaysia. The early studies provided some preliminary data and laid the foundation for the first-ever serological survey of CAE among small ruminants in the state of Selangor, where 8.8% of 91 goats tested positive for CAEV (Jesse *et al*. 2018). These studies gave insights for further studies using a larger sample size to elucidate the overall seroprevalence and risk factors of the disease. This study was therefore conducted to investigate the seroprevalence and contributing factors of CAEV among sheep and goats in the two coastal states of Peninsular Malaysia.

## MATERIALS AND METHODS

### Ethics Statement

The Institutional Animal Care and Use Committee of Universiti Putra Malaysia (UPM/IACUC/AUP-013/2018) and the Department of Veterinary Services (DVS) approved all sample and data collection in Negeri Sembilan and Terengganu states.

### Study Area

Terengganu state is on the east coast of Peninsular Malaysia between 5.3117° N, 103.1324° E of the equator. The state has a total area of 13,035 km^2^ with 1.125 million human population and the vegetation is typically tropical rainforest climate characterised by high temperature and high humidity all year round. Before the discovery of oil and gas reserves in the state in 1974, agriculture was the mainstay of its economy. Negeri Sembilan state is on the southwest coast of the Peninsular Malaysia between 2.8 2.7258° N, 101.9424° E. The state has an estimated total area of 6,686 km^2^ with 1.098 million human populations. The mainstay of economy of the state is rubber and oil palm plantations agriculture in addition to manufacturing and tourism. The state of Terengganu has 37,000 small ruminants while Negeri Sembilan has 63,673 small ruminants kept in individual smallholder or government farms found at the fringes of forest vegetations in rural localities [Department of Veterinary Services (DVS) 2018].

The predominant breed of goats in Malaysia includes indigenous Katjang and imported Jamnapari, Anglo Nubian, Alpine, Saanen, Toggenburg, Australian Feral and the Boer. The sheep breeds include the local Malin and exotic White Dorper, Dorset Horn, Wiltshire, Suffolk, Romney, Commercial Merino/Border Leicester crosses, Barbados Black Belly and Santa Ines (Chandrawathani 2004). With very few exceptions, the smallholder farm is a semi-intensive system which allows daytime grazing and provisions of housing and feed supplementation at night.

### Sample Size

The estimation of the sample size for herd level true prevalence of CAE in the study area was calculated using EpiTools^®^ statistical software according to Humphry *et al*. (2004) based on the assumptions of 95% confidence interval (CI), 5% desired absolute precision, 100% specificity, 99% sensitivity, and 8.42% assumed true prevalence as reported by Jesse *et al*. (2018). The specified minimum number of samples was 128 animals, but 262 animals were randomly sampled from seven different farms to increase precision. The availability of samples and farmers cooperation determined the total number of samples collected from each farm.

### Study Design and Sampling

The targeted smallholder farmers were contacted through Department of Veterinary Services (DVS) in Terengganu and Negeri Sembilan states for informed consent to take part in the study. Farms included in this survey practiced either intensive management, which encloses animals to pens without grazing or semi-intensive management, which allowed restricted grazing of animals during the day with provisions for housing and feed supplementation at night. Seven farms that agreed to take part in the survey were visited to collect blood samples, individual animal records and management data. The animals were aged by examining their dental eruption pattern and grouped them as young (less than one year) or adult (one year and above), and classified the various breeds were identified according to their phenotypic characteristics. Approximately 5 mL of blood sample was collected from apparently healthy animals in plain vacutainer tubes by jugular venipuncture after applying adequate physical restraint. The animal biodata was collected on a sampling form and each farmer completed a questionnaire on farm management practices.

### Serum Extraction and Storage

Serum was separated from coagulated blood by centrifugation at 3000 revolutions per minute (rpm) for 20 min (Eppendorf^®^ AG 22331, Hamburg Germany) and kept frozen at −20°C before ELISA.

### Detection of Serum Antibody by ELISA

The Qayee-Bio caprine arthritis encephalitis virus (CAE) sandwich-ELISA test kit with a sensitivity of 100% and specificity of 99.6% (QY-E140077) was used for detection of specific antibodies according to the manufacturer’s instructions (RuiHua Network Technology Co., LT, China). The optical densities (OD) was measured at 450 nm using ELISA Microplate Reader (Tecan Sunrise^®^, Switzerland) and the percentage inhibitions calculated as 100 × [1–(Sample optical density/Negative control O.D.)] were interpreted as follows: OD values ≥ 40% = positive, OD values ≤ 30% = negative and OD values 30%–40% = doubtful.

### Statistical Analysis

Statistical analysis involved the initial entry and summary of all data in Microsoft^®^ Excel Spreadsheet Program Version 2016 followed by Chi-square univariable analysis using the IBM^®^ Statistical Package for Social Sciences (SPSS) software version 25.0 to calculate the apparent prevalence and the associations between the serological status of small ruminants and epidemiological variables. Further analysis involved the calculation of true prevalence according to Rogan and Gladen (1978) and their 95% confidence intervals, according to Brown *et al*. (2001) using EpiTools^®^ software version 0.5–10.1. Lastly, a Backward Conditional Multivariable Logistic Regression analysis was done using IBM SPSS version 22.0 to determine the odds ratio associated with risk factors of CAEV among sheep and goats at 95% CI and a 5% level of significance.

## RESULTS

The results of this survey have revealed 21.4% (95% CI: 15.8–28.6) apparent and 20.6% (95% CI: 14.5–27.8) true individual seroprevalence, and 85.7% herd-level seroprevalence for CAEV. Our results further show that the seroconversion rate was significantly (*p* < 0.05) higher among animals in Negeri Sembilan (52.4%) than Terengganu state (7.2%). A similar pattern was also observed among the farms whereby a significantly higher prevalance (*p <* 0.05) was recorded in farms F (56.6%) and G (44.8%) in Negeri Sembilan compared to farms A (12.5%), B (10%), D (8.0%) and C (6.7%) in Terengganu where all animals in farm E were tested negative. Breed-wise, the prevalence of CAEV (*p <* 0.05) was significantly higher in Boer goats (44.8%) than the Kajang goats (20.2%) and Barbados Black Belly sheep (10.0%). Based on the system of production, the seroprevalence of CAE (*p <* 0.05) was significantly higher in meat (27.4%) than mixed (27.4%) and dairy (1.8%) animals. On the other hand, the seroprevalence of CAE (*p >* 0.05) was insignificantly higher among goats (23.4%) than sheep (10%), but there was no difference in prevalence between the different age group (*p* > 0.05) and sexes (*p* > 0.05). Similarly, there was an insignificant difference (*p* > 0.05) in prevalence between intensive (24.6%) and semi-intensive (17.7%) management system of small ruminants (Table 1). Multivariable association between the significant risk factors (states, breed and production type) and serological status further revealed that sheep and goats in Negeri Sembilan state were more-likely (OR = 10.424, 4.887–22.237; *p* < 0.05) at risk of CAEV than those in Terengganu (Table 2).

## DISCUSSION

Small ruminant lentivirus infections such as CAEV interferes with the growth and welfare of affected sheep and goats (Tavella *et al*. 2018). The impact of CAE on well-being and performance of small ruminants affects their productivity and cause economic losses to the farmer (Nagel-Alne *et al*. 2014). This study has revealed 21.4% (95% CI: 15.8–28.6) apparent and 20.6% (95% CI: 14.5–27.8) seroprevalence of CAEV among small ruminants, which is higher than a preliminary study that reported a 8.42% seroconversion rate among goats from Selangor, Malaysia (Jesse *et al*. 2018), but is comparable to a previous survey by Al-Qudah *et al*. (2006) who reported a 23.2% seroconversion rate among goats in Jordan. However, contrary to our result, higher individual seroconversion rates have been previously reported in Taiwan (Yang *et al*. 2016) and the United States (Cutlip *et al*. 1992; Jones 2014). A previous study by Jesse *et al*. (2018) ascribed the observed differences in reported seroprevalence rates to the peculiarities in climate, sample size, and the diagnostic sensitivity and specificity of test kits. Furthermore, the 85.7% herd level seroprevalence of CAE in this study is higher than the 73% reported by Cutlip *et al*. (1992) in the US, 71% reported by Ghanem *et al*. (2009) in Somalia and 31% reported by Lin *et al*. (2011) in Thailand. But our result is lower than 98.5% herd seroprevalence reported by Yang *et al*. (2016) in Taiwan. The observed difference in seroprevalence of CAEV in different parts of the world may be due to different ecological and management factors of small ruminants. Generally, CAEV is more common among goats in most industrialised countries which practiced intensive management due to ease of transmission through colostrum, iatrogenic route and prolonged direct physical contact with infected hosts (Tu et *al*. 2017).

This study detected a significant difference in the seroprevalence of small ruminant CAEV among goats in the two states with ten-times more likelihood of seropositivity in Negeri Sembilan state. This finding shows an unequal distribution of small ruminant CAEV in different parts of the country and agrees with the results of an earlier study in Malaysia which reported a lower prevalence in Selangor (Jesse *et al*. 2018). The higher prevalence of CAEV among small ruminants in Negeri Sembilan may be because the farms are more organised into an intensive management where animals are mostly confined. Moreover, previous reports indicate that discrepancy in the seroprevalence of CAEV within and between countries are connected to the difference in diagnostic sensitivities of laboratory methods due to delayed seroconversion (Rimstad *et al*. 1993), fluctuations or loss of antibodies (Barquero *et al*. 2013; Tu *et al*. 2017) and genetic heterogeneity of regional virus strains (Tu *et al*. 2017). The observed difference in seroprevalence rates of CAEV infection between different farms in the two states may be due to different farm management practices. There are variations in intensive management practices which increase the risk of CAEV such as pooled colostrum/milk feeding, stocking density, shared farm equipment such as needles, ear tag applicator, wool sharers and milking machines among different farms (Tu *et al*. 2017). Moreover, the different farms sourced breeding stock from different sources and have different levels of biosecurity.

Earlier studies have linked species difference in seroprevalence of anti-CAEV antibodies to variations in species susceptibility, viral characteristics, management practices and other sundry factors (Barquero *et al*. 2011). Although CAEV affects all small ruminants (Tu *et al*. 2017), the infection and corresponding antibody response may show different patterns in sheep and goats, and account to differences in reported seroconversion and diagnostic outcomes (Barquero *et al*. 2013). The significantly higher seroprevalence of CAEV among exotic Boer goats in this study agrees with the earlier report by Jesse *et al*. (2018). Our result further concurs with other reports which showed that different breeds of small ruminants vary in their susceptibility to CAEV infection (Rowe & East 1997; Adams *et al*. 1983; Greenwood 1995; Lin *et al*. 2011). The observed breed difference in the prevalence of CAEV in the current study may be explained by genetically determined factors which regulate resistance and or susceptibility to diseases (Thrusfield 2005).

In contrast to previous studies which detected gender-linked differences in the seroprevalence of CAEV among goats in Australia (Grewal *et al*. 1986), Thailand (Ratanapob *et al*. 2009) and Malaysia (Jesse *et al*. 2018), no sex difference in CAEV seroconversion rate occurred in this study. This finding suggests an equal chance of exposure and susceptibility of male and female small ruminants examined in this study due to similar management and environmental conditions. Furthermore, our result is different from previous studies in Somalia (Ghanem *et al*. 2009), Thailand (Ratanapob *et al*. 2009; Lin *et al*. 2011) and others (Cutlip *et al*. 1992; Al-Qudah *et al*. 2006) where older goats were reportedly more likely to be CAEV seropositive than younger ones. Epidemiological evidence shows that CAEV may indiscriminately affect all age groups of small ruminants in a flock (Al-Ani & Vestweber 1984) due to multiple transmission routes (Tu *et al*. 2017).

The significantly higher seroprevalence of CAE seen among goats raised for meat production in this study is contrary to earlier reports that CAE is more prevalent among dairy goats (Greenwood 1995; Rowe & East 1997). The discrepancy in findings could be because most of the goats sampled in this study are kept for meat production and farmers in this area seldom keep large numbers of dairy goats. Furthermore, the insignificantly higher prevalence of CAE among intensively raised small ruminants in the present study agrees with Brinkhof *et al*. (2010) who reported that CAE is a significant problem of the modern intensive management system. Our result further agrees with Lin *et al*. (2011) who reported higher prevalence of CAE among intensively managed goats in Thailand. Intensive management practices such as the stocking density, flock size, age of weaning and the conditions of housing, hygiene and ventilation (de la Concha-Bermejillo 1997; Pérez *et al*. 2010; Leginagoikoa *et al*. 2010) are contributing factors of CAEV within flocks (Barquero *et al*. 2013). Moreover, some intensive practices such as pooled milk/colostrum feeding, needle reuse and sharing of milking equipment among others are significant factors for virus transmission within flocks (Grewal *et al*. 1986; Tu *et al*. 2017).

## CONCLUSION AND RECOMMENDATION

The high frequency of anti-CAEV antibodies among the small ruminant herds under study supplies further evidence for the prevalence of caprine arthritis encephalitis virus in Peninsular Malaysia and underscores the role of active serological surveillance in disease control. The different breeds, production systems, farms and states of small ruminants expressed different patterns of CAEV seroconversion. The emergence of CAE in Peninsular Malaysia is a potential threat to the small ruminant industry and developing agricultural economy. Further studies to elucidate the genetic characteristics, distribution and risk factors of CAEV should be carried out to plan a holistic program of prevention and control in Malaysia. Eradication of CAE is a difficult task, but its economic impacts can be reduced by lowering the herd prevalence through effective herd health programs. Pasteurisation of milk, serological monitoring, culling of the infected animals, artificial insemination and sterilisation of milking or other farm equipment are practical measures to lower herd infection and keep productivity. Moreover, the enforcement of strict biosecurity and quarantine measures will strengthen prevention efforts due to the dependence of the Malaysian livestock industry on the importation of breeding stock.

## Figures and Tables

**Table 1:** The apparent and true prevalence of CAEV among sheep and goats in two states of Malaysia.

Variables	Categories	Apparent prevalence	True prevalance (%)	95% CI	*p-*value
States	Negeri Sembilan	43/82 (52.4%)	52.0	38.6–65.9	0.000*
	Terengganu	13/180 (7.2%)	6.3	3.6–13.9	
Farms	A	5/40 (12.5%)	11.6	4.3–31.4	0.000*
	B	4/40 (10%)	9.1	3.0–28.4	
	C	2/30 (6.7%)	5.7	1.3–27.7	
	D	2/25 (8.0%)	7.1	1.6–32.0	
	E	0/45 (0.0%)	−1.0	−1.0–6.9	
	F	30/53 (56.6%)	56.2	39.3–72.4	
	G	13/29 (44.8%)	44.3	24.3–67.3	
Species	Sheep	4/40 (10.0%)	9.1	3.0–28.4	0.057
	Goat	52/222 (23.4%)	22.7	16.9–31.5	
Breed	SH-Barbados Black	4/40 (10.0%)	9.1	3.0–28.4	0.002*
	Belly				
	GT-Katjang	39/193 (20.2%)	19.4	13.8–28.6	
	GT-Boer	13/29 (44.8%)	44.3	24.3–67.3	
Gender	Male	20/95 (21.1%)	20.3	12.4–33.5	0.924
	Female	36/167 (21.6%)	20.8	14.5–30.8	
Age	Young	21/90 (23.3%)	22.6	13.9–36.4	0.576
	Adult	35/172 (20.4%)	19.5	13.6–29.3	
Production	Meat	54/197 (27.4%)	26.7	20.1–36.2	0.000*
	Dairy	1/55 (1.8%)	0.83	0.21–13.8	
	Mixed	1/10 (10%)	0.91	0.49–39.8	
Management	Semi-intensive	22/124 (17.7%)	16.9	10.6–28.1	0.174
	Intensive	34/138 (24.6%)	23.9	16.5–35.1	
Overall		56/262 (21.4%)	20.6	15.8–28.6	

*Note*:

* Significant (*p* < 0.05),

CI = Confidence Interval.

**Table 2:** Multivariable association between putative risk factors and CAEV seropositivity.

Variables	Categories	β	SE	*p*-value	AOR (95%CL)
States	Terengganu				1.00 (Reference)
	Negeri Sembilan	2.344	0.387	0.000*	10.424 (4.887, 22.237)

*Note*: *β* = Regression coefficient, SE = Standard Error,

* Significance,

AOR = Adjusted Odds Ratio, CI = Confidence Interval.
